# Some Improvements in the Method of Local Analgesia

**Published:** 1903-10

**Authors:** 


### ABSTRACTS.

### Some Improvements in the Method of Local Analgesia.

Arthur E. J. Barker, F. R. C. S., Eng., in a clinical lecture at University College, London, published in *The Lancet* for July 25, 1903, said among other things that several points must be borne in mind, among them the mechanical and physical difficulties in infiltrating all the nerves supplying an extensive field of operation. To inject the whole area so as to reach all its nerves would mean in many cases the use of much more of the toxic drug than is necessary, and in some cases so much as to be dangerous.

The author refers to certain observations by Braun, of Leipsic, on a method of overcoming the drawbacks incident to the usual mode of producing local anesthesia. This method is based upon the old experience that anything which retards or diminishes the circulation of the blood in a part enhances the potency of the analgesic agent. Experiments were made with adrenalin, a very small quantity of which was injected with B-eucaine (or cocaine) into the author's own arm, and subsequently into the arms of numerous patients. After the lapse of twenty minutes the part was quite blanched and wholly insensitive to pain, remaining so for about two hours. Adrenalin, alone, used in this way had no analgesic effect, while the results of the use of the combined solutions of B-eucaine and adrenalin were far superior to those produced by B-eucaine alone.

The most convenient way to prepare the solution is as follows: powders each containing 0.2 gramme (3 grains) of B-eucaine and 0.8 gramme (12 grains) of pure sodium chloride are kept in thick glazed paper, ready for use. When needed one powder is dissolved in 100 c.c. (3½ fluid ounces) of boiling distilled water, and 1 c.c. of Parke, Davis & Co.'s solution adrenalin chloride is added when the fluid is cool. The solution is left in the Jena glass beaker in which it has been boiled, which is carefully covered and placed in a vessel of warm water to keep it at blood heat. The injection is made by means of a simple syringe of glass and metal of 10 c.c. capacity, with rubber washers, which can be sterilised by boiling.

To illustrate his method the author describes in detail the performance of an operation for the radical cure of inguinal hernia. The hernia is first reduced and the index finger is thrust into the external ring as far as possible. Along this finger the needle is entered and the inguinal canal is filled with 10 c.c. of the solution. An endeavor is made to inject it all around the neck of the sac so as to reach the genital branch of the genitocrural nerve. The needle is then entered at the external end of the line of incision in the skin, and is made to infiltrate the superficial layers of the latter down to the root of the scrotum, making the resulting wheal at least an inch longer at each end than the incision is to be. Injections are then made at a point half an inch to the inner side of the anterior superior spine of the ilium, the needle being thrust towards the ilioinguinal nerve, and at a point about one inch above the middle of Poupart's ligament where the iliohypogastric nerve is most conveniently met. Then the thigh is flexed and another syringeful is injected along the ramus of the pubis and the root of the scrotum or labium.

It is necessary to wait twenty minutes after the last injection for the full effect of the adrenalin to develop. The whole field of operation should be blanched and insensitive to pricks but not to touch,—analgesia, not anesthesia. The incision may then be made with confidence that no pain will be felt. The absence of oozing of blood is noticed. Only large vessels bleed at all.

Success depends upon a mastery of the principles, and practice in the details of the method. It is not enough to inject the fluid under the skin generally. Due regard must be had to the position and course of the nerves supplying the structures to be dealt with. The adrenalin compound, by slowing the circulation through the part prevents the anesthetic agent from being rapidly washed away. The writer has used this method in thirty operations including the radical cure of hernia, strangulated hernia, orchidectomy, removal of varicose veins, psoas abscess, loose body in knee, tumor of neck (actinomycosis), colotomy, Thiersch skin grafting, and cystic adenoma of the thyroid.

